# Supplementary material for: Modelling cigarette smoke-induced lung vascular dysfunction using an alveolus-on-chip
Source: Mater Today Bio. 2026 Jul 6;39:103429. doi: 10.1016/j.mtbio.2026.103429 (PMC13380759; doi:10.1016/j.mtbio.2026.103429)
Supplement: Multimedia component 1 [file mmc1.docx]

**Supplementary material**

**Modelling cigarette smoke-induced lung vascular dysfunction using an alveolus-on-chip**

Abilash Ravi^1*^, Tarek Gensheimer^2*^, Annemarie van Schadewijk^1^, Xinhui Wu^3,4,5^, Jill R. Johnson^5^, Martin C. Harmsen^3,6^, Reinoud Gosens^3,4^, Pieter S. Hiemstra^1^, Andries D. van der Meer^2#^ and Anne M. van der Does^1#^

^*^Shared first authors

^#^Shared last authors

**Table S1. List of primary and secondary antibodies used for staining**

| **S.No** | **Gene** | **Forward primer (5’-3’)** | **Reverse primer (5’-3’)** |
| --- | --- | --- | --- |
| 1 | *CD31* | AAATGCTCTCCCAGCCCAGGAT | GCAACACACTGGTATTCGACGTCTT |
| 2 | *LYVE1* | GGGTTGGAGATGGATTCGTGG | ATAGGCTGCAAACTGTCGGC |
| 3 | *CLDN5* | CTCTGCTGGTTCGCCAACAT | CAGCTCGTACTTCTGCGACA |
| 4 | *PRPRB* | TACCAATGGATCAACAGTGCC | GCATCAGCCGGTATCGTTCC |
| 5 | *GPIHBP1* | GCAACCTGACGCAGAACTG | CCAGGGTGGGACATTGCAC |
| 6 | *EDNRB1* | GTCCCAATATCTTGATCGCCAG | AAGGCACCAGCTTACACATCT |
| 7 | *ATP5B* | TCACCCAGGCTGGTTCAGA | AGTGGCCAGGGTAGGCTGAT |
| 8 | *RPL13A* | AAGGTGGTGGTCGTACGCTGTG | CGGGAAGGGTTGGTGTTCATCC |
| 9 | *NG2* | GCCACGTTGTCAGTCGATG | CCCATAGGGGACCTCTAGGG |

| **S.No** | **Antibody** | **Supplier** | **Catalog** | **Species** | **Dilution** |
| --- | --- | --- | --- | --- | --- |
| 1 | CD31 | Dako | #M0823 | mouse | 1:200 |
| 2 | Neuron-glial antigen 2 (NG2) | Abcam | #ab255811 | rabbit | 1:800 |
| 3 | pro-surfactant protein C | Milllipore | #ab3786 | rabbit | 1:100 |
| 4 | Cleaved caspase-3 | Cell signaling | #9664 | rabbit | 1:400 |
| 5 | Periaxin | Sigma Aldrich | #HPA001868 | rabbit | 1:100 |
| 6 | AF488 donkey anti-mouse | Thermo Fischer Scientific | #A-21202 | donkey | 1:400 |
| 7 | AF647 donkey anti-rabbit | Thermo Fischer Scientific | #A-31573 | donkey | 1:400 |
| 8 | Mouse IgG1 isotype control | R&D systems | #MAB002 | mouse | 1:200 |
| 9 | Rabbit IgG isotype control | Biotechne | #NBP2-24891 | rabbit | 1:100 |

**Table S2. List of forward and reverse primers used for qPCR analysis**

**Supplementary methods:**

*Conditioned medium transfer experiment*

Alveolar epithelial type-2 cells (AEC2) were seeded (100.000 cells/insert) on inserts and cultured in Science Cell alveolar medium added with CHIR99021 (4 µM; Merck LifeScience) and A83-01 (5 µM; Tocris) medium until confluency was reached (~4 days). After reaching confluency, A83-01 was removed from the medium. Next apical medium was removed and insert cultures were subsequently exposed to whole cigarette smoke (WCS) as described in the main manuscript methods section. After a total of 15 min. of exposure and ventilation, basal medium was removed and new medium was added to both the basal (600 µl) and apical (150 µl) compartment. 24h later, apical medium and basal medium were removed and transferred to chips with a vascularized hydrogel (day 5 of culture), cultured according to the methods in the main manuscript. Each chip received 100 µl conditioned medium in the apical compartment of the chip culture from either the apical or basal insert compartment after which they were placed back in the incubator on the rocking platform. AEC2 inserts received new basal medium, were exposed to WCS again, after which basal medium was removed again, and both apical and basal medium were renewed. This procedure was repeated up to 7 consecutive days, i.e. WCS exposures. Each day vascular integrity was assessed using brightfield microscopy, but no differences could be observed (N=3).

**Supplementary figure legends:**

**Fig S1. (A)** Flow cytometry analysis of endothelial cells isolated from lung tissue and expanded at P1, for CD140b/PDGRFβ and CD31 (n=3 different donors). **(B)** Bright field images (of n=3 different donors) depicting morphology of endothelial monolayers expanded at P1. **(C)** Vascular network formation of endothelial cells isolated from COPD donor. Representative confocal image (n=2) after 6 days of self assembled vascular network stained with endothelial cell marker, CD31 (in green) and pericyte marker NG2 (in red) and nuclei with DAPI stain (in blue). Scale bar 70 µm. (**D**) Flow cytometry analysis of endothelial cells inefficiently isolated with mesenchymal contamination, from lung tissue and expanded at P1, for CD140b/PDGRFβ and CD31 (n=1). (**E**) Bright field images (of n=1) depicting morphology of endothelial monolayers in the presence of mesenchymal cells, expanded at P1. **(F)** Representative confocal image after 6 days of self-assembled vascular network (n=3 different donors) stained with pericyte marker NG2 (in red) and nuclei with DAPI stain. Scale bar 70 µm.

**Fig S2. Gene expression analysis of pericyte monolayer. (A)** qPCR analysis of mRNA expression in pericytes (*n*=1) and endothelial cells (*n*=1) for markers of endothelial cells (*CD31*), macrovascular endothelium (*LYVE1*), microvascular endothelium (*CLDN5*), gCAP-microvascular endothelium (*GPIHBP1*, *PTPRB*), alveolar repair marker (*BMP6*), aCAP-microvascular endothelium (*EDNRB1*), and pericyte marker (NG2).

**Fig S3.** Representative fluorescent image of a non-perfusable vascular network characterised by the accumulation of 2µm fluorescent beads (blue) at the pillar of the inlet, indicating obstruction and lack of bead entry into the network. Scale bar 150 μm

**Fig. S4.** Representative confocal image (n=3 different donors) of an air-exposed alveolus-on-chip with continuous vascular network (**A**) and a WCS-exposed alveolus-on-chip with disrupted vascular networks (**B;** unmatched) marked with endothelial marker, CD31 (in green), pericyte marker, NG2 (in red) and AEC2 monolayer in the middle stained for pro-SFTPC (in red). Scale bar, 25 µm.

**Supplementary video legends:**

**Video S1.** Representative confocal video (n=3 different donors) after 6 days of a self-assembled vascular network formation on chip with endothelial cells in green (CD31), pericytes in red (NG2), and nuclei in blue (DAPI). Scale Bar, 50 µm.

**Video S2.** Representative fluorescent video of self-assembled vascular network perfused using fluorescent beads (blue, 2 µm diameter). Scale bar, 100 µm.

**Video S3.** Representative fluorescent video of self-assembled vascular network (red - phalloidin) perfused using fluorescent beads (blue, 2 µm diameter) and live THP-1 monocytes (green). Scale bar, 150 µm.

**Video S4.** Representative confocal video (n=3 different donors) of alveolus on-chip with self-assembled vascular network stained for CD31 (in green) and AEC2 monolayer stained for (pro-SFTPC) (in red) and nuclei stained with DAPI (in blue). Scale bar: 20 µm.
