# Supplementary material for: Modelling cigarette smoke-induced lung vascular dysfunction using an alveolus-on-chip
Source: Mater Today Bio. 2026 Jul 6;39:103429. doi: 10.1016/j.mtbio.2026.103429 (PMC13380759; doi:10.1016/j.mtbio.2026.103429)
Supplement: Multimedia component 2 [file mmc2.pdf]

Figure S1

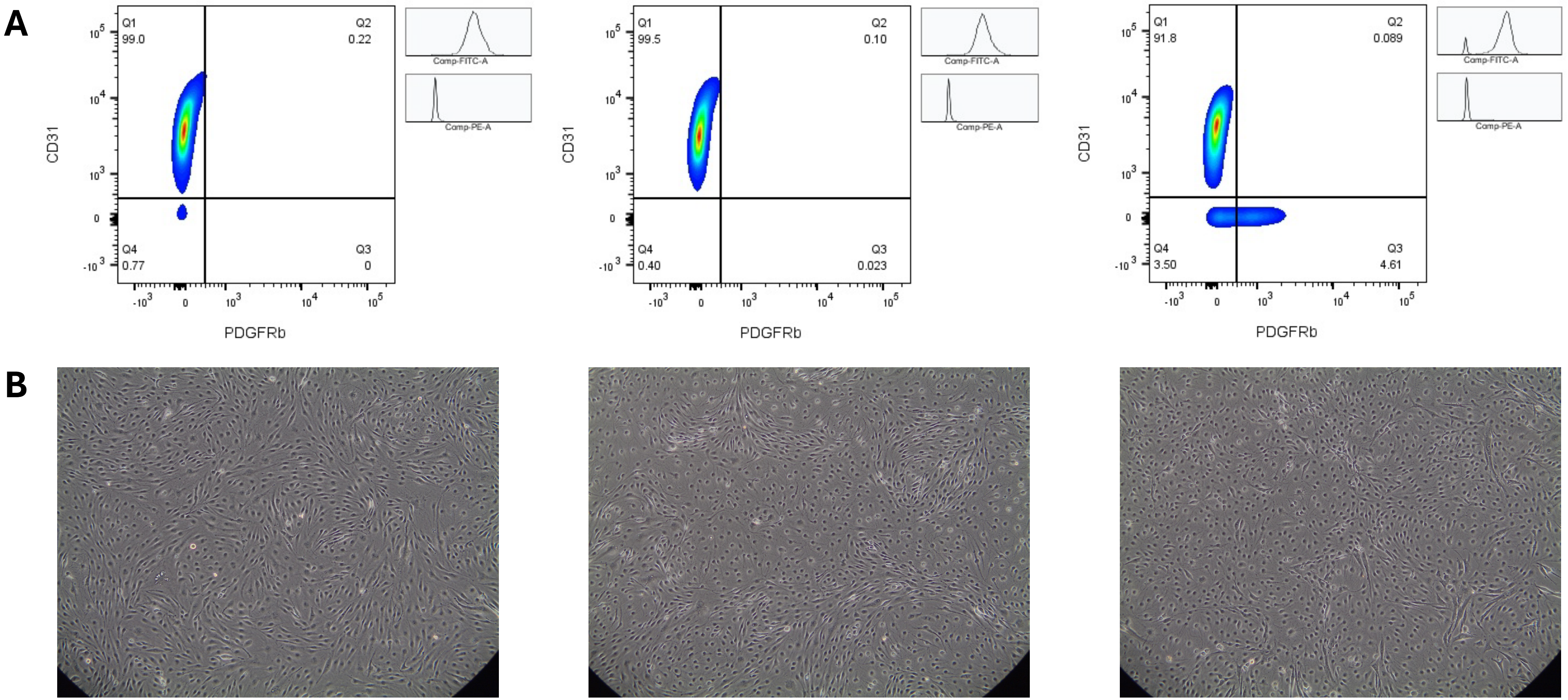

Figure S1. (**A**) Flow cytometry analysis of endothelial cells isolated from lung tissue and expanded at P1, for CD140b/PDGRFβ and CD31 (n=3 different donors). (**B**) Bright field images (of n=3 different donors) depicting morphology of endothelial monolayers expanded at P1.

Figure S1

C

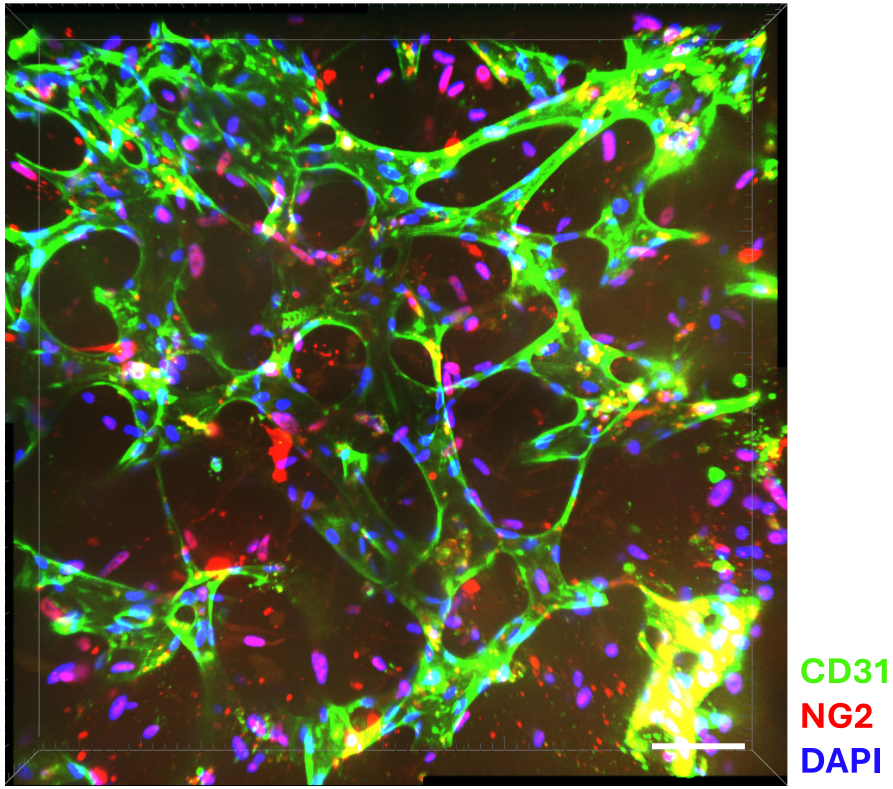

Figure S1. (C) Vascular network formation of endothelial cells isolated from COPD donor. Representative confocal image (n=2) after 6 days of a self-assembled vascular network stained with endothelial cell marker, CD31 (in green) and pericyte marker NG2 (in red) and nuclei with DAPI stain (in blue). Scale Bar, 70 µm.

Figure S1

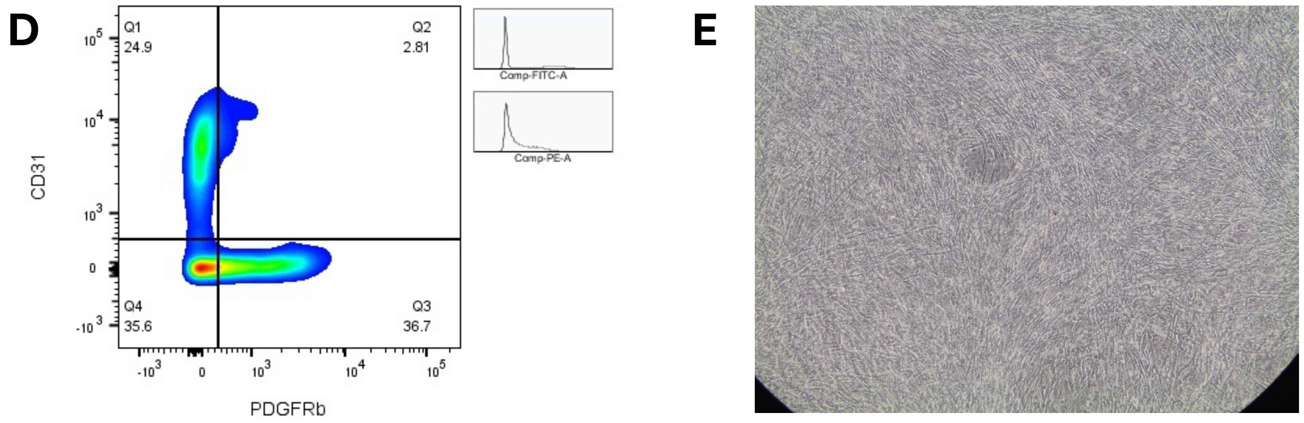

Figure S1. (**D**) Flow cytometry analysis of endothelial cells inefficiently isolated with mesenchymal contamination, from lung tissue and expanded at P1, for CD140b/PDGRFβ and CD31 (n=1). (**E**) Bright field images (of n=1) depicting morphology of endothelial monolayers in the presence of mesenchymal cells, expanded at P1.

Figure S1

F

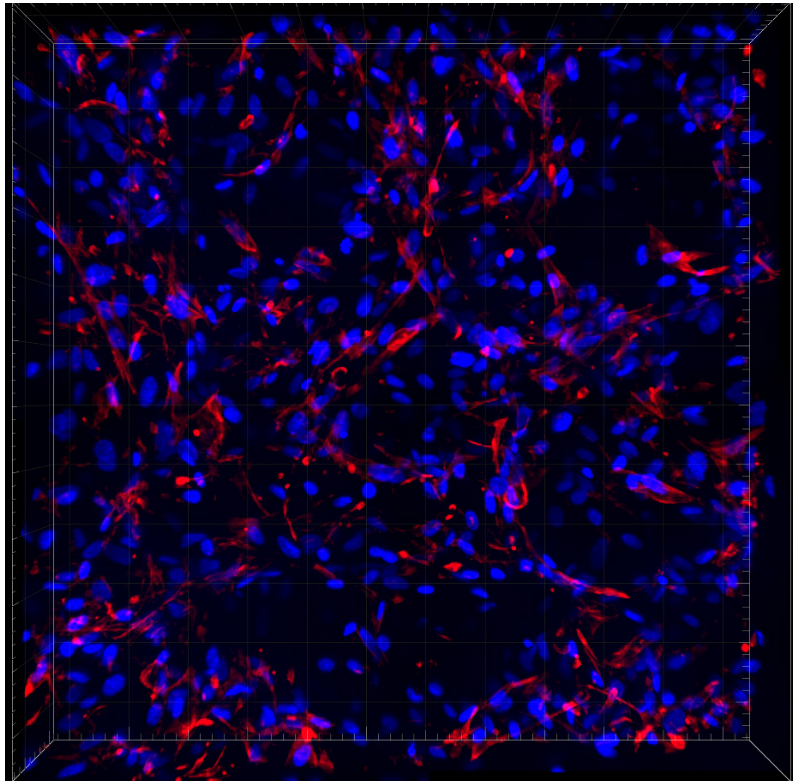

Figure S1. (F) Pericytes are embedded within the endothelial networks. Representative confocal image after 6 days of a self-assembled vascular network (n=3 different donors) stained with pericyte marker NG2 (in red) and nuclei with DAPI stain (in blue). Scale Bar, 70 µm.

Figure S2

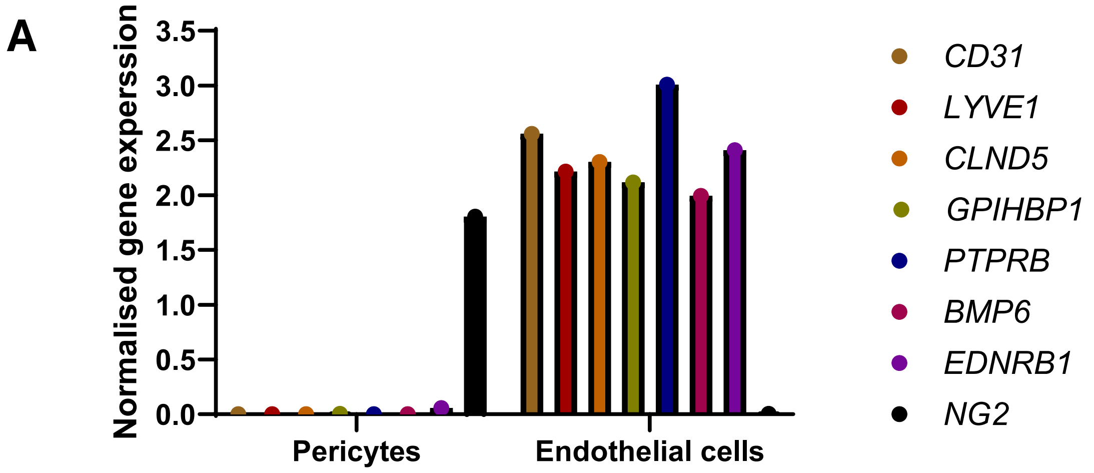

Figure S2. Gene expression analysis of pericyte monolayer. (A) qPCR analysis of mRNA expression in pericytes (*n*=1) and endothelial cells (*n*=1) for markers of endothelial cells (*CD31*), macrovascular endothelium (*LYVE1*), microvascular endothelium (*CLDN5*), gCAP-microvascular endothelium (*GPIHBP1*, *PTPRB*), alveolar repair marker (*BMP6*), aCAP-microvascular endothelium (*EDNRB1*), and pericyte marker (NG2).

Figure S2

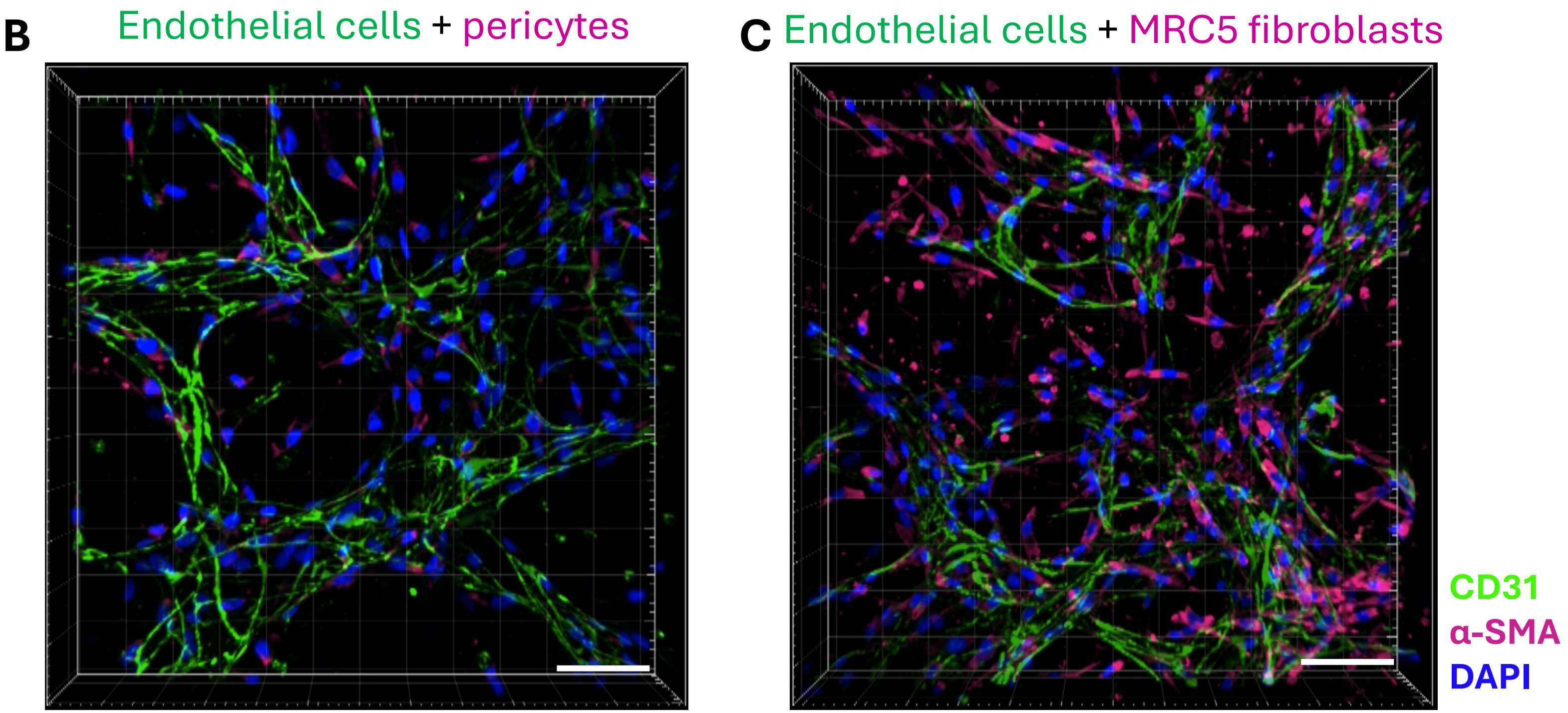

Figure S2. Comparison of endothelial network formation with pericytes and fibroblasts. Representative confocal image after 6 days of endothelial vascular network formation with pericytes **(B)** and MRC5 fibroblasts **(C)** stained for endothelial cell marker CD31 (in green) and in α-SMA (in magenta), marker for both pericyte and fibroblasts and nuclei with DAPI stain (in blue). Scale Bar, 20 μm.

Figure S3

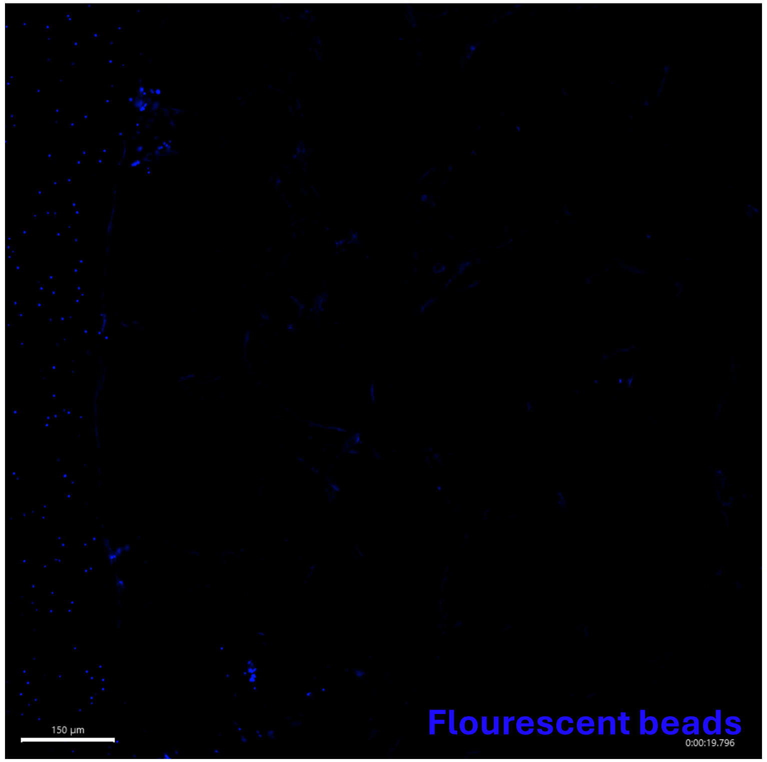

Figure S3. Representative fluorescent image of a non-perfusable vascular network, characterized by the accumulation of 2 µm fluorescent beads (blue) at the pillar in the inlet, indicating obstruction and lack of bead entry into the network. Scale bar, 150 µm

Figure S4

A B

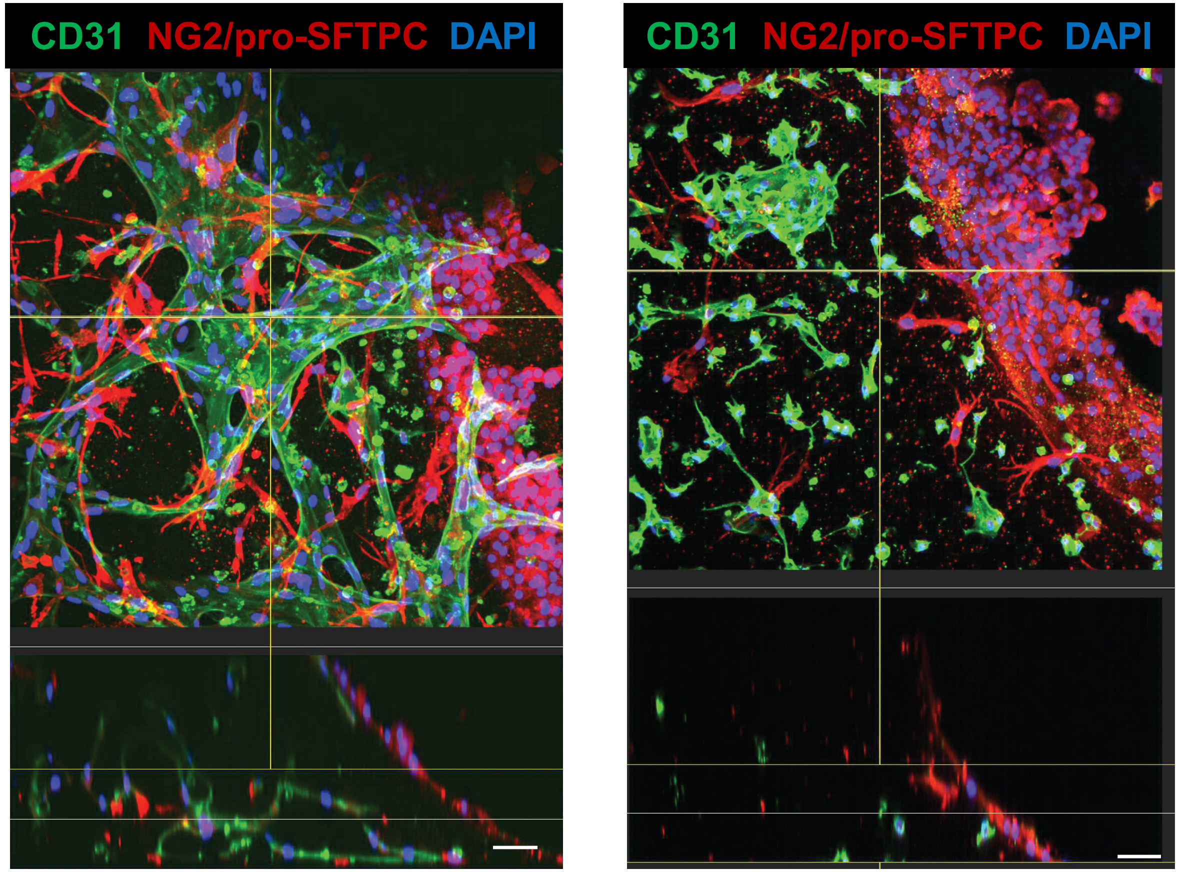

AIR + AEC2 WCS + AEC2

Fig. S4. Representative confocal image (n=3 different donors) of an air-exposed alveolus-on-chip with continuous vascular network **(A)** and a WCS-exposed alveolus-on-chip with disrupted vascular networks **(B;** unmatched with A**)** marked with endothelial marker, CD31 (in green), pericyte marker, NG2 (in red) and AEC2 monolayer in the middle stained for pro-SFTPC (in red). Scale bar, 25 µm.
